# Diagnostic performance of ultrasensitive rapid diagnostic test for the detection of *Plasmodium falciparum* infections in asymptomatic individuals in Kisangani, Northeast Democratic Republic of Congo

**DOI:** 10.1186/s12936-023-04790-1

**Published:** 2023-11-19

**Authors:** Mbumba Lupaka, Teshome Degefa, Kasahun Eba, Ahmed Zeynudin, Delenasaw Yewhalaw

**Affiliations:** 1 Faculty of Medicine and Pharmacy, University of Kisangani, Kisangani, Democratic Republic of the Congo; 2 School of Medical Laboratory Sciences, Institute of Health, Jimma University, Jimma, Ethiopia; 3 Department of Environmental Health Sciences and Technology, Institute of Health, Jimma University, Jimma, Ethiopia; 4 Tropical and Infectious Diseases Research Center (TIDRC), Jimma University, Jimma, Ethiopia

**Keywords:** Asymptomatic malaria, Alere™ Malaria Ag P.f usRDT, *Plasmodium falciparum*, Democratic Republic of Congo

## Abstract

**Background:**

Ultrasensitive rapid diagnostic test (usRDT) was recently developed to improve the detection of low-density *Plasmodium falciparum* infections. However, its diagnostic performance has not been evaluated in the Democratic Republic of Congo (DRC). This study aims to determine the performance of the usRDT in malaria diagnosis in asymptomatic individuals under field condition in Kisangani, Northeast of DRC.

**Methods:**

A community-based cross-sectional study was carried out from June to August 2022 on 312 asymptomatic individuals residing in the city of Kisangani. Capillary blood samples were collected by finger prick for microscopic examination of thick and thin blood film, RDTs, and nested polymerase chain reaction (PCR). Alere™ Malaria Ag P.f usRDT and conventional RDT (cRDT/SD Bioline Malaria Ag P.f) kits were used for the detection of *Plasmodium* histidine rich protein 2 (HRP2) antigen as a proxy for the presence of *P. falciparum*. The diagnostic performance of the usRDT was compared with cRDT, microscopy and PCR.

**Results:**

The prevalence of asymptomatic *P. falciparum* malaria was 40.4%, 42.0%, 47.1% and 54.2% by cRDT, microscopy, usRDT and PCR, respectively. By using PCR as a reference, usRDT had sensitivity and specificity of 87.0% (95% CI 81.4–91.7) and 100.0% (95% CI 97.5–100.0), respectively, whereas the cRDT had sensitivity and specificity of 74.6% (95% CI 67.3–80.9) and 100% (95% CI 97.1–100.0), respectively. By using microscopy as a reference, usRDT had sensitivity and specificity of 96.9% (95% CI 92.4–99.2) and 89.0% (95% CI 83.5–93.1), respectively, while the cRDT had sensitivity and specificity of 96.2% (95% CI 92.3–98.7) and 100% (95% CI 97.9–100.0), respectively.

**Conclusion:**

The usRDT showed better diagnostic performance with higher sensitivity than the cRDT which is currently in use as point-of-care test. Further research is necessary to assess the access and cost-effectiveness of the usRDTs to use for malaria surveillance.

## Background

Malaria remains a global public health concern, affecting hundreds of millions of people each year. In 2021 alone, an estimated 247 million cases and 619,000 deaths due to malaria were reported globally. Most of the cases (95%) and deaths (96%) were in the African region [1]. The Democratic Republic of Congo (DRC) is one of the highest burden African countries, accounting for 12% of all malaria cases and 13% of malaria-related deaths globally [1]. It is estimated that about 97% of the population of the DRC live in zones with stable malaria transmission [2]. *Plasmodium falciparum* is the predominant cause of malaria in the country [3].

Vector control using long-lasting insecticidal nets (LLINs) and indoor residual spraying (IRS), and prompt detection and treatment of symptomatic malaria cases are the key strategies widely used for malaria prevention and control in Africa [4]. The scale-up of these control tools and strategies have significantly reduced malaria burden in the region in the past decades, averting an estimated 663 million malaria cases between 2000 and 2015 [4, 5]. Although this progress has stalled since 2015 due to different reasons [6, 7], the past gains have brought the notion of malaria elimination in Africa [8], and countries are intensifying the control interventions to keep up the momentum of fighting against malaria.

One of the major challenges to malaria control and elimination efforts in sub-Saharan Africa is asymptomatic malaria, which is highly prevalent in many African settings including DRC [9–12]. Asymptomatic individuals often do not seek treatment for their infection but represent a potential reservoir of malaria parasites, thereby sustaining the transmission [13–15]. The World Health Organization (WHO) recommends transforming malaria surveillance into a core intervention to address this challenge and hasten the progress towards elimination [8]. This involves using active case detection (ACD) methods such as mass or targeted screening of population following an index case identified at a health facility to treat asymptomatic malaria-positive cases and eventually interrupt the transmission [8, 16]. ACD is usually performed in the community and hence depends on prompt diagnosis by using rapid diagnostic tests (RDTs) and microscopy [8, 16, 17].

However, the effectiveness of ACD is limited due to the inability of the commonly used malaria diagnostic tools to detect low density asymptomatic infections [17–20]. Microscopic examination of thick and thin blood film is considered the gold standard method as it allows identification of parasite species and estimation of parasitaemia [21]. However, lack of competent microscopists and misdiagnosis due to low parasitaemia are the main challenges in many countries [21]. Conventional RDTs (cRDTs) have also played a significant role in malaria control efforts, with additional advantages in terms of speed, cost-effectiveness and convenience of use with minimum training. However, they have low sensitivity with detection limit of about 100–200 parasites/µL (p/µL) and hence they cannot detect low-density *P. falciparum* infections [20, 22–24]. Molecular techniques such as polymerase chain reaction (PCR) have greater sensitivity than both the cRDTs and microscopy, with a capacity of detecting infections with parasite density as low as 0.2 p/µL [25–27]. However, using PCR in the field on large scale is impractical as it requires expensive laboratory equipment, reagents and well-trained personnel [25, 28].

To overcome the limitation of the cRDTs, ultra-sensitive RDT (Abbott Alere™ Malaria Ag *P. falciparum* usRDT) has recently been developed for the detection of *P. falciparum* infections [24]. It is a histidine rich protein 2 (HRP2)-based test that has more than tenfold lower limit of detection (10–40 pg/ ml of HRP2) when compared to the conventional SD Bioline Malaria Ag P.f RDT (800–1000 pg/ml of HRP2), and was prequalified by the WHO in 2019 [29]. The usRDT has a capacity of detecting low-density infections with parasitaemia as low as 1.2 p/µL, with a potential of detecting new erythrocytic-stage *P. falciparum* infections in earlier days than the cRDT [24, 29, 30].

Previous studies conducted in different malaria transmission settings have shown wide variation in usRDT sensitivities compared to PCR [24, 29, 31–33]. Cross-sectional surveys conducted in Mozambique, Uganda, Myanmar and Ghana reported sensitivity estimates of usRDT ranging from 68.2% to 84% for detection of asymptomatic *P. falciparum* infections [24, 30, 34], and sensitivity of up to 99% for detection of infections among febrile patients [35, 36]. In systematic reviews, the pooled sensitivity of the usRDTs in asymptomatic individuals is estimated to reach up to 50% versus 27% for cRDTs when PCR was used as a reference [37, 38]. In some studies where malaria prevalence was determined by using both usRDT and cRDT, the prevalence was found to be 1.5–2 times higher using the usRDT compared to the cRDT [33, 39–42]. On the other hand, usRDT did not show better diagnostic performance over the cRDT in some studies [31, 32].

Although most studies from other countries reported better sensitivity of usRDTs compared to the cRDT, no study evaluating the diagnostic performance of usRDTs has been conducted in the DRC. Hence, this study aimed to evaluate the performance of the usRDT in diagnosing *P. falciparum* malaria under field condition among asymptomatic individuals in Kisangani, Northeast of DRC.

## Methods

### Study design and setting

**Fig. 1:**
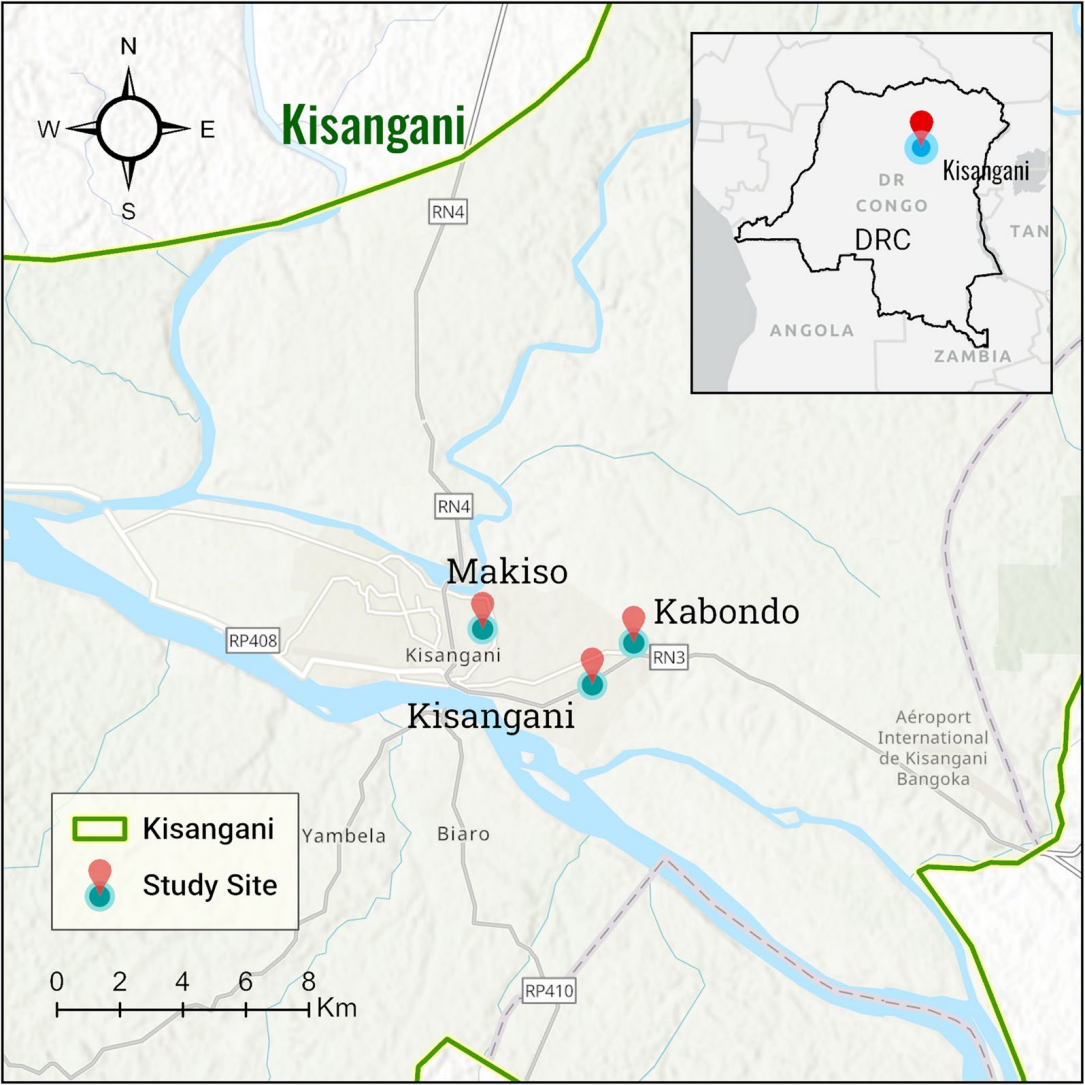
Map of the study area

A community-based cross-sectional study was carried out in selected communes of Kisangani from June to August, 2022. Kisangani is a capital city of the province of Tshopo in the Northeast of the DRC (Fig. 1). It is located at 1724 km northwest of Kinshasa, the capital of DRC. The city has an area of 1910 Km^2^ and the estimated population size is 1602,144 [43]. The climate of Kisangani is equatorial type in general. The temperature varies between 25° and 28°, and it rains all year round with periods of maximum and minimum rain. The city is made up of 6 communes, each of which constitutes a health zone and includes a general reference hospital as well as health centers. Malaria is endemic in the province of Tshopo in general and the city of Kisangani in particular. *Anopheles mosquitoes* are known to maintain an intense (up to 1000 infective bites per person per year) and perennial transmission of malaria in the area [2, 44].

### Sample size and sampling techniques

The sample size was calculated using the method of Buderer et al*.* [45]. First, the proportion of true positive (TP) plus false negative (FN) for sensitivity (Se); and the true negative (TN) plus false positive (FP) for specificity (Sp) were determined using the following equations:

$$TP+FN= {Z}_{\frac{\alpha }{2}}^{2}\frac{Se \left(1-Se\right)}{{W}^{2}}$$

$$TN+FP= {Z}_{\alpha /2}^{2}\frac{Sp (1-Sp)}{{W}^{2}}$$

where ${Z}_{\alpha /2}$, the normal distribution value, was set to 1.96 as corresponding with the 95% confidence interval (CI), W, the maximum acceptable width of the 95% CI, is set to 7%, and the expected sensitivity of 68.2% and specificity of 99% are defined based on the estimates from previous studies done in Mozambique [34]. The next step was to calculate the sample size (N) required for sensitivity and specificity using the following equations:

$$N\left(Se\right)=\frac{TP+FN}{P}$$

$$N\left(Sp\right)=\frac{TN+FP}{P}$$

where P is the anticipated prevalence of malaria. Taking the prevalence of asymptomatic malaria of 63% in 2016 in the northeast part of DRC [9], and considering an expected nonresponse rate of 15%, a minimum of 312 and 15 samples were required for sensitivity and specificity, respectively. Finally, the larger sample size (312) was used for this study.

**Fig. 2:**
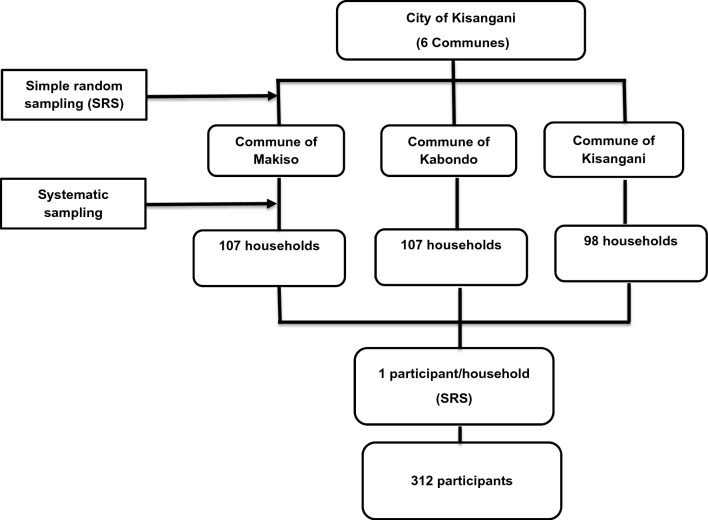
Flow chart of the study participant selection

The city of Kisangani is administratively subdivided into six communes. This study covered three communes selected randomly: Kabondo, Makiso and Kisangani. The sample size was proportionally allocated to each commune based on their population size. A systematic sampling technique was used to select 312 households, and one individual was randomly selected per household (Fig. 2). Households were visited during the weekends and evening hours when most individuals were at their home to make sure that each household member has equal chance of being selected as a study participant. When the randomly selected individuals were not found at home during the first household visits, return visits were scheduled to collect samples and relevant data from the selected individuals. Individuals who took anti-malarial treatment within four weeks of the blood sample collection were not included in this study.

### Data collection

Information on socio-demographic factors, malaria history, and preventive methods was obtained from the participants using a pre-tested, structured questionnaire. The questionnaire included questions on sex, age, educational level, residence, occupation, marital status, history of fever in the preceding 72 h, previous anti-malarial treatment in the preceding month, and whether the participants slept under insecticide-treated nets (ITNs)/LLINs the previous night. Prior to commencement of the study, a pretest was conducted in a neighboring commune on five percent of the total sample size, and the questionnaire was further modified after the pretest. The axillary temperature was measured using an electronic thermometer before each blood sample collection and fever was defined as a temperature ≥ 37.5 °C. Finger-prick blood sample was collected from each study participant for RDTs, microscopic examination of thick and thin blood film, and PCR. For the PCR, 2–3 dry blood spots (DBS), each with 50µL of blood, was prepared on Whatman 3 MM filter paper for each study participant. Blood sample collection from all study participants was done by experienced laboratory technologists.

### Laboratory work

#### Rapid diagnostic tests

Both cRDT (SD Bioline Malaria Ag P.f, Catalog Number: 05FK50-40, Abbott, USA) and usRDT (Alere™ Malaria Ag P.f Ultra-Sensitive, Catalog Number: 05FK140, Abbott, USA) were used to test for *P. falciparum* HRP2 according to the manufacturer’s instructions. For both RDT kits, 5 µL of capillary whole blood was applied to sample well, followed by addition of four drops of assay buffer. The test results were read after 15 min for the cRDT and after 20 min for the usRDT. A positive signal (red color) on both control and test lines was considered as a positive test result for *P. falciparum*, and a positive control line without signal on the test line was interpreted as a negative result. Any test result without a signal on the control line was considered an invalid test and the test was repeated in this case. All individuals found positive for malaria by RDTs were advised to visit the nearest health facility for treatment.

#### Microscopic examination

Thick and thin blood smears were made on the same slide, and the thin smears were fixed by absolute methanol. The slides were then transported to the Kisangani provincial laboratory and stained with 10% Giemsa following standard operating procedures [46]. All slides were examined microscopically by an experienced laboratory technologist. The slides were considered negative if no parasite was seen after examining 100 high power fields. Asexual parasite density was quantified against 200 leucocytes. This was converted to the number of parasites per microliter of blood assuming a white blood cell (WBC) count of 8,000/µL as recommended by the WHO [21]. Parasite density was classified as low parasitaemia (< 1000 p/µL), moderate (1000–4999 p/µL), high (5000–99,999 p/µL), and hyper parasitaemia (≥ 100,000 p/µL) [47]. Quality control slides (all positive blood smear slides and 10% of the negative slides) were re-examined by another blinded senior laboratory technologist.

#### Molecular diagnosis using polymerase chain reaction

The collected DBS were transported to the Institut National de Recherche Biomédicale (INRB) in Kinshasa to test for the presence of *P. falciparum* by PCR. DNA was extracted from the DBS using the QIAamp DNA Mini Kit (QIAGEN, UK), following the manufacturer’s instructions.

A nested PCR was used to amplify species-specific sequences of the small sub-unit ribosomal ribonucleic acid (18S SSU rRNA) genes of *P. falciparum* as previously described elsewhere [48, 49]. In the first amplification reaction, a pair of oligonucleotide primers (genus-specific rPLU1: 5′-TCAAAGATTAAGCCATGCAAGTGA-3′ and rPLU5: 5′-CCTGTTGTTGCCTTAAACTCC-3′) was used to amplify fragment of the genes [49]. The product of this first reaction was then used as DNA template for the second amplification reaction. Both the first and second PCR amplifications were carried out in a total volume of 25 µL consisting of 2 µL template; 0.25 μM of each primer; 1.5 mM magnesium chloride; 0.2 mM dNTPs; 1X PCR Buffer; and 1U Taq polymerase. Primers specific to *P. falciparum* (rFAL1: 5′-TTAAACTGGTTTGGGAAAACCAAATATATT-3′ and rFAL2: 5′-ACACAATGAACTCAATCATGACTACCCGTC-3′) were used [49]. The PCR assays were performed using a PTC-100 Thermal Cycler (MJ Research Inc.), with the following cycling condition: initial denaturation at 94 °C for 4 min, followed by 25 cycles of denaturation at 94 °C for 1 min, annealing at 65 °C for 2 min and extension at 72 °C for 2 min, and final extension at 72 °C for 4 min. The amplicons were electrophoresed using 2% agarose gel prepared in Tris–borate-EDTA and stained with ethidium bromide for visual detection by ultraviolet transilluminator. Purified 3D7 DNA parasite strain was used as a positive control whereas a negative control without a DNA template was run in all reactions for quality control. *Plasmodium falciparum* infections detected by PCR but not by microscopy were classified as sub-microscopic infections [50].

### Data analysis

Data were checked for completeness and consistency, coded and entered into Epi-info version 7.0, and exported into the Statistical Package for Social Science version 25 (SPSS, Chicago, IL, USA) and JASP 0.16 (JASP TEAM, University of Amsterdam, The Netherlands) for further analysis. The basic characteristics of the study participants were summarized using descriptive statistics.

Sensitivity, specificity, positive predictive value, and negative predictive value of cRDT and usRDT were determined using microscopy and PCR as reference methods [51]. The 95% confidence interval was calculated for each performance indicator using MedCalc statistical software [52]. Kappa coefficient was calculated to assess the agreement among the different diagnostic methods. Depending on the value of the kappa, the level of the agreement between RDTs and the reference methods was considered non-existent if the kappa value was between 0 and 0.2; minimal if it was between 0.21 and 0.39; low between 0.40 and 0.59; moderate between 0.60 and 0.79; strong between 0.80 and 0.90 and almost perfect agreement with a kappa value greater than 0.90 [53].

## Results

### Characteristics of the study participants

**Fig. 3:**
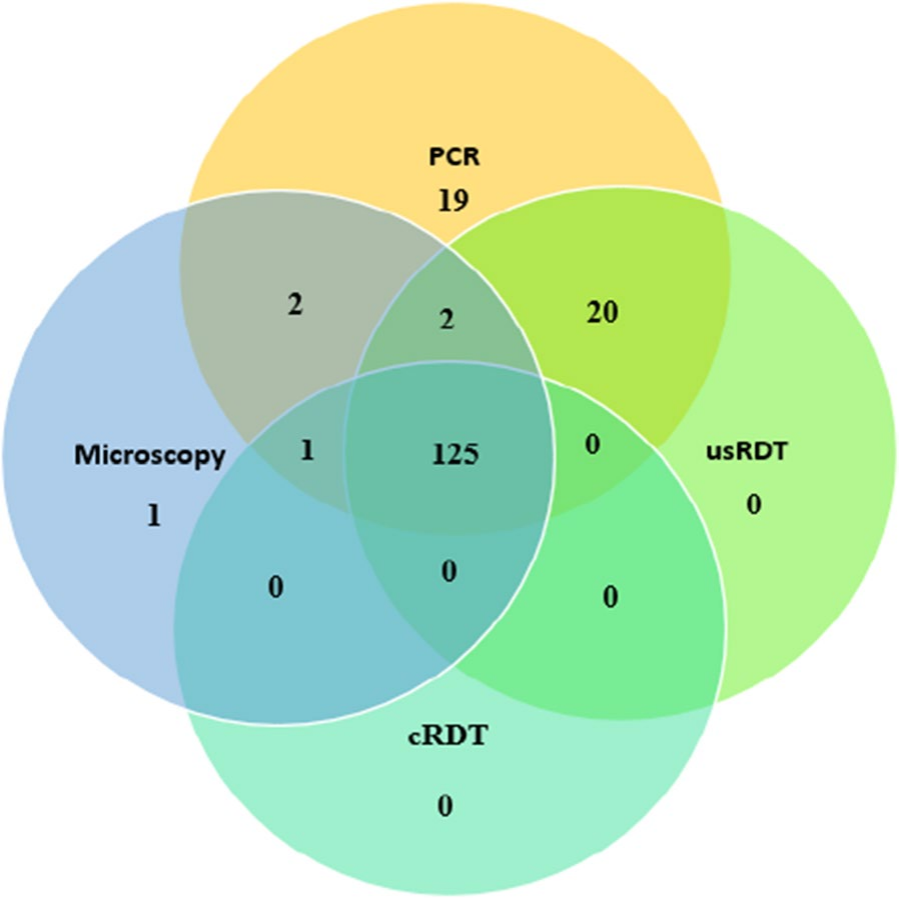
Venn diagram showing *P. falciparum* positivity among asymptomatic individuals by different diagnostic methods in Kisangani, DRC

Most of the study participants (94.2%) were over the age of 15 years, with mean age of 25.4. The majority (54.5%) of the participants were females. Study participants from Makiso, Kabondo and Kisangani represent 34.4%, 34.3% and 31.3%, respectively. Most (94%) of the study participants reported that they had at least one LLIN in their house and 79% of them reported to have used LLINs the previous night. Only 4.2% of the study participants reported that IRS was applied to their houses in the past 6 months. Malaria prevalence by cRDT, microscopy, usRDT and PCR was 40.4%, 42.0%, 47.1% and 54.2%, respectively (Table 1). Of the 312 study participants tested, 125 (40.1%) were positive for the 4 diagnostic methods used in this study (Fig. 3).

**Table 1:** Prevalence of asymptomatic *P. falciparum* infection by diagnostic test among asymptomatic individuals in Kisangani, DRC

Diagnostic test	Frequency (n = 312)	Percentage
cRDT	126	40.4
usRDT	147	47.1
Microscopy	131	42.0
PCR	169	54.2

### RDTs performance against microscopy

Using microscopy as a reference, usRDT showed an overall sensitivity and specificity of 96.9% (95% CI 92.4–99.2) and 89.0% (95% CI 83.5–93.1), respectively. The cRDT had sensitivity and specificity values of 96.2% (95% CI 91.3–98.7) and 100% (95% CI 97.9–100.0), respectively. The level of agreement between microscopy and both RDT types was significant, with a strong agreement documented between usRDT and microscopy (Kappa = 0.84, p < 0.01), and almost perfect agreement between cRDT and microscopy (Kappa = 0.96, p < 0.01) (Table 2).

**Table 2:** Performance of cRDT and usRDT against microscopy for detection of *P. falciparum* among asymptomatic individuals in Kisangani, DRC

Parameters	cRDT (n = 312)	usRDT (n = 312)
True positive	126	127
False positive	0	20
True negative	181	161
False negative	5	4
Sensitivity % (95% CI)	96.2 (91.3–98.7)	96.9 (92.4–99.2)
Specificity % (95% CI)	100 (97.9–100.0)	89.0 (83.5–93.1)
PPV % (95% CI)	100 (97.1–100.0)	86.4 (80.7–90.6)
NPV % (95% CI)	97.3 (93.9–98.8)	97.6 (93.9–99.1)
Kappa % (p-value)	96.7 (< 0.01)	84.5 (< 0.01)

*PPV* Positive predictive value, *NPV* negative predictive value, *CI* confidence interval, *cRDT* conventional RDT, *usRDT* ultrasensitive RDT

### Performance of RDTs against PCR

The cRDT failed to detect a total of 43 of the 169 positive cases by PCR, representing a false negative rate of 25.4%. Of these 43 cases, 22 were correctly detected by usRDT. In comparison, the usRDT failed to detect *P. falciparum* in 22 of the 169 positive cases, giving a false negative rate of 13.0% (Fig. 3). Using PCR as a reference, the usRDT showed higher sensitivity (87.0%, 95% CI 81.4–91.7) when compared with cRDT (74.6%, 95% CI 67.3–80.9). Both cRDT and usRDT were highly specific (100%). Overall, the level of agreement between both cRDT and usRDT and PCR was significant (p < 0.05). The level of agreement between usRDT and PCR was stronger (Kappa = 0.86, p < 0.01) and higher than that of cRDT versus PCR (Kappa = 0.72, p < 0.01), which was a moderate agreement (Table 3).

**Table 3:** Performance of cRDT and usRDT against PCR for detection of *P. falciparum* among asymptomatic individuals in Kisangani, DRC

Parameters	cRDT (n = 312)	usRDT(n = 312)
True positive	126	147
False positive	0	0
True negative	143	143
False negative	43	22
Sensitivity % (95% CI)	74.6 (67.3–80.9)	87.0 (81.4–91.7)
Specificity % (95% CI)	100 (97.5–100.0)	100 (97.5–100.0)
PPV % (95% CI)	100 (97.1–100.0)	100 (97.5–100.0)
NPV % (95% CI)	76.9 (72.0–81.1)	86.7 (81.5–90.6)
Kappa % (p-value)	72.0 (< 0.01)	86.0 (< 0.01)

*PPV* Positive predictive value, *NPV* negative predictive value, *CI* confidence interval, *cRDT* conventional RDT, *usRDT* ultrasensitive RDT

### Distribution of parasitaemia according to RDTs positive results

Most of the malaria positive study participants (97.0%) had low parasitaemia whereas 3.0% of them had moderate parasitaemia. The mean parasitaemia was 541.30 (± 300.32) p/µL for cRDT positive results with minimum and maximum values of 78 p/µl and 2678 p/µL, respectively. Regarding the usRDT positive results, the mean parasitaemia was 467.62 (± 334.62) p/µL with a minimum parasitaemia of 0 p/µL and a maximum parasitaemia of 2678 p/µL.

### Performance of RDTs based on the parasite density

The performance of the two RDT kits in terms of sensitivity was higher in people with moderate parasitaemia compared to people with low parasitaemia. For the usRDT, the sensitivity was 100% and 86.6% (95% CI  80.4–91.4) for moderate and low parasitaemia cases, respectively, whereas for the cRDT, the sensitivity was 100% and 73.8% (95% CI  66.4–80.3) for moderate and low parasitaemia cases, respectively (Table 4).

**Table 4:** Performance of cRDT and usRDT by parasite density among *P. falciparum* infected asymptomatic individuals in Kisangani, DRC

Parameters		cRDT	usRDT
Low parasite density (< 1000 p/µL), n = 164	Sensitivity % (95% CI)	73.8 (66.4–80.3)	86.6 (80.4–91.4)
	Specificity % (95% CI)	100 (97.5–100.0)	100 (97.5–100.0)
Moderate parasite density (1000–4999 p/µL), n = 5	Sensitivity % (95% CI)	100 (-)	100 ()
	Specificity % (95% CI)	–	–

*cRDT* conventional RDT, *usRDT* ultrasensitive RDT, *n* number of samples, *CI* confidence interval

## Discussion

In this study, the usRDT showed better diagnostic performance in detecting asymptomatic *P. falciparum* infections with sensitivity of 87% as compared to the cRDT which had sensitivity of 74.6%, using PCR as a reference. This is consistent with the findings of previous studies conducted in Uganda and Myanmar [24], Ghana [30], Mozambique [34] and Haiti [54], which reported higher sensitivity for the usRDT than the cRDT. The level of agreement between the usRDT and PCR was stronger and higher than that of cRDT versus PCR. This is likely due to the fact that usRDT has a detection limit closer to PCR than the cRDT [24, 30, 49].

On the other hand, usRDT yielded false negative results in 13.0% of the PCR positive samples although this was two times lower when compared to the false negative rate of the cRDT (26.0%). The false negative results recorded by both usRDT and cRDT might be due to the presence of low parasite density below their respective limit of detection. The presence of parasites with *P. falciparum* HRP2/3 gene deletions could also result in false negative RDT results [55–57], although the magnitude of the HRP2/3 gene deletions was not determined in this study. Moreover, a prozone effect due to an excess of either antibodies or HRP2 antigens could contribute to the false negative RDT results [58].

Studies have shown that HRP2 antigen of *P. falciparum* can persist in the circulation for weeks after an infection is cleared by treatment, and this can give false positive RDT results even in the absence of active infection [59, 60]. In this study, however, both usRDT and cRDT showed 100% specificity with no false positive results by both RDT types, using PCR as a reference. The absence of false positive RDT result might be due to participants selection criteria implemented during this study as people who took anti-malarial drugs within four weeks of the commencement of sample collection were not included in the study. With microscopy as a reference, the usRDT had a similar sensitivity but lower specificity than the cRDT. The difference in specificity between usRDT and cRDT documented in this study was similar to the previous reports from Uganda and Myanmar [24]. The lower specificity of usRDT could be partly attributed to the enhanced sensitivity of the usRDT at detecting lower density parasitaemia and lower concentrations of HRP2 antigens compared to the cRDT [24, 29, 32, 61, 62] and partly due to the presence of sub-microscopic infections. In this study, 23% of the malaria positive cases were submicroscopic infections and half of these were detected by the usRDT, while cRDT missed all submicroscopic PCR positive cases (Fig. 3).

The relative sensitivity of the two RDT types varied based on parasite density. The usRDT exhibited significantly higher sensitivity than the cRDT in detecting low parasitaemia infections, whereas both usRDT and cRDT showed 100% sensitivity for moderate parasitaemia. Other studies have also documented similar findings [24, 29]. In Uganda and Myanmar for instance, the usRDT was shown to detect low parasitaemia infections with parasite density as low as 1.2 p/µL, while cRDT failed to detect *P. falciparum* in samples with parasitaemia of up to 183 p/uL [24], confirming that the usRDT has a better diagnostic performance in detecting low-density *P. falciparum* infections than the cRDT. This suggests that the usRDT could be a potential diagnostic tool for screening asymptomatic and submicroscopic *Plasmodium* infections for prompt case treatment especially in settings targeted for malaria elimination.

It is worth mentioning that the prevalence of asymptomatic *P. falciparum* infection was high, around 54.2% by PCR, in the study area despite high LLIN ownership. A multi-center study conducted in 2016 in DRC by Mvumbi et al*.* has also reported an overall malaria prevalence of 43% using PCR, and a prevalence rate of over 63% in Maniema region near Tshopo district, where the city of Kisangani is located [9]. Thus, the high asymptomatic malaria prevalence documented in this study could be due to the study setting since it is located in an area with intense malaria transmission where the number of infectious mosquito bites can reach as high as 1000 bites per person per year [44]. The high malaria parasite prevalence despite high LLIN coverage in the study area suggests that further studies should be carried out to assess LLIN utilization, as sub-optimal utilization could affect the effectiveness of the LLINs. On the other hand, people could be bitten by mosquito vectors in the early evening and morning hours before people sleep under the LLINs, suggesting the need to determine vector biting behaviour in the study area. Moreover, determining insecticide susceptibility level of malaria vectors and other contributing factors [63] is crucial in order to design appropriate control interventions. It is also important to note that the prevalence of sub-microscopic asymptomatic *P. falciparum* infections was 12.5% in the study area. This implies that malaria surveillance system should be strengthened in the area using sensitive diagnostic tools such as usRDT in order to accurately identify malaria hotspot areas to prioritize control intervention based on evidence.

The limitation of this study is that parasitaemia was quantified using microscopy and parasite density was not determined for PCR positive samples which were negative by microscopy.

## Conclusion

In this study, usRDT showed higher sensitivity than cRDT in detecting *P. falciparum* infections among asymptomatic individuals in a high malaria transmission setting of Kisangani. This is the first study that evaluated the performance of usRDT in DRC and generated evidence to guide policy and the program on the implementation of this tool to diagnose asymptomatic individuals. This study also documented high prevalence of asymptomatic *P. falciparum* infections among communities residing in the city of Kisangani. Further research is necessary to assess the impact and cost-effectiveness of the usRDTs to be used in the health care system.

## Data Availability

Data supporting the conclusions of this article are included within the article. Raw data are available from the corresponding author upon reasonable request.

## Abbreviations

ACD: Active case detection
CI: Confidence interval
cRDT: Conventional rapid diagnostic test
DBS: Dry blood spot
DRC: Democratic Republic of the Congo
HRP2/3: Histidine rich protein 2/3
IRS: Indoor residual spraying
LLIN: Long-lasting insecticidal net
PCR: Polymerase chain reaction
RDT: Rapid diagnostic test, Se: sensitivity
Sp: Specificity
usRDT: Ultrasensitive rapid diagnostic test
WHO: World Health Organization
